# Mycobiome analysis of leaf, root, and soil of symptomatic oil palm trees (*Elaeis guineensis* Jacq.) affected by leaf spot disease

**DOI:** 10.3389/fmicb.2024.1422360

**Published:** 2024-12-06

**Authors:** Abiodun Abeeb Azeez, Daniel Ofeoritse Esiegbuya, Adebola Azeez Lateef, Fred O. Asiegbu

**Affiliations:** ^1^Department of Forest Sciences, University of Helsinki, Helsinki, Finland; ^2^Rainforest Research Station, Forestry Research Institute of Nigeria, Ibadan, Nigeria; ^3^Pathology Division, Nigerian Institute for Oil Palm Research (NIFOR), Benin City, Nigeria; ^4^Department of Plant Biology, Faculty of Life Sciences, University of Ilorin, Ilorin, Nigeria

**Keywords:** oil palm, leaf spot disease, mycobiome, fungal community, symptomatic, asymptomatic

## Abstract

Recently, attention has been shifting toward the perspective of the existence of plants and microbes as a functioning ecological unit. However, studies highlighting the impacts of the microbial community on plant health are still limited. In this study, fungal community (mycobiome) of leaf, root, and soil of symptomatic leaf-spot diseased (SS) oil palm were compared against asymptomatic (AS) trees using ITS2 rRNA gene metabarcoding. A total of 3,435,417 high-quality sequences were obtained from 29 samples investigated. Out of the 14 phyla identified, Ascomycota and Basidiomycota were the most dominant accounting for 94.2 and 4.7% of the total counts in AS, and 75 and 21.2% in SS, respectively. *Neopestalotiopsis* is the most abundant genus for AS representing 8.0% of the identified amplicons compared to 2.0% in SS while *Peniophora* is the most abundant with 8.6% of the identified amplicons for SS compared to 0.1% in AS. The biomarker discovery algorithm LEfSe revealed different taxa signatures for the sample categories, particularly soil samples from asymptomatic trees, which were the most enriched. Network analysis revealed high modularity across all groups, except in root samples. Additionally, a large proportion of the identified keystone species consisted of rare taxa, suggesting potential role in ecosystem functions. Surprisingly both AS and SS leaf samples shared taxa previously associated with oil palm leaf spot disease. The significant abundance of *Trichoderma asperellum* in the asymptomatic root samples could be further explored as a potential biocontrol agent against oil palm disease.

## Introduction

Oil palm is a monocotyledonous crop and a prominent member of the family Arecaceae (Syn. Palmae). *Elaeis guineensis* is indigenous to the tropical rainforest zones of West Africa (Maluin et al., 2020). Globally, it is recognized as the most productive oil crop and a vital source of vegetable oil (Swaray et al., 2020). It accounts for approximately 8 million hectares of agricultural land and yield per unit area is relatively higher than the other 16 crops known as fat and oil-producers (Mayes, 2003; Okolo et al., 2019; Swaray et al., 2020). Production of oil palm is projected to be around 240 million tons by the year 2050 (Barcelos et al., 2015). The current world’s top-five oil palm-producing countries are Indonesia, Malaysia, Thailand, Colombia, and Nigeria (Voora et al., 2019). However, across all the regions where oil palm is cultivated, diseases remain one of the serious challenges to its sustainable productivity (Chung, 2012; Pornsuriya et al., 2013) which is important to meet the increasing demand for its products, especially biofuel, chemical, and food ingredients (Kirkman et al., 2022; Murphy, 2014).

Leaf spot is predominantly a fungal disease affecting oil palm in many areas where it is grown. Aetiologically, leaf spots become leaf blight usually at the later stage of infection when numerous spots fuse and manifest as an aggregate mass on an affected leaf (Elliott, 2005). This disease is especially more challenging in the cultivation of the Tenera variety known to be more productive (Pornsuriya et al., 2013). Oil palms at the nursery (usually seedlings stage, more than 3 months in age) or juvenile stage are more susceptible to this infection (Elliott, 2005; Turner, 1981). Many historical incidences of oil palm leaf spot and leaf blight attributed to different causal agents have been reported from various geographical regions (Turner, 1981). These include the catastrophic destruction of oil palm plantations by anthracnose caused by *Glomerella cingulata*, *Botryodiplodia palmarum*, and *Melanoconium* sp. in Africa, and *Curvularia* leaf blight caused by *Curvularia eragrostidis* in Asia (Aderungboye, 1977). Leaf spot disease also affects other plant species such as coconut, and severity varies according to region (Turner, 1981). The non-host-specific *Pestalotiopsis* leaf spot caused by *Pestalotiopsis* spp. is prevalent in all palm-growing regions and has a peculiar ability to attack any part of the leaf (Elliott, 2006; Elliott, 2009; Turner, 1981). *Cercospora* leaf spot caused by *Cercospora elaeidis* is a common foliar disease of nursery seedlings and juvenile oil palms in West Africa (Rajagopalan, 1973).

Microorganisms often constitute the microbiome including bacteria, fungi, archaea, and protists as well as viruses inhabit different parts and organs of terrestrial plants forming a close association or network with one another and the hosts. The resulting host-microbiome interactions underpin the health and fitness in plants and largely determine the expression of a plethora of important traits. Particularly functional roles of fungal biota in host growth, disease resistance, nutrient mobilization, stress tolerance, synthesis of phytohormones, and water and gas exchange between plants and the environment (Friesen et al., 2011; Kovalchuk et al., 2018; Lyu et al., 2021; Turner et al., 2013; Vorholt, 2012) have been reported. The term “mycobiome” was coined from combining “mycology” and “microbiome” to describe the fungal community within a specific environment. Since its first use in 2010, its appearance in publications has significantly increased, reflecting substantial progress in the field (Ghannoum et al., 2010). This progress can be attributed to the critical roles of fungal communities in various environments, from human health and fitness (such as the gut and lung mycobiomes) (Zhang et al., 2022) to agricultural ecosystems (Kirkman et al., 2022) and beyond. However, there is still limited information on the factors that determine fungal and other microbiome compositions in plant communities. Plant mycobiome and other microbes are heterogeneous in nature and their composition and structure are under the influence of intrinsic factors (host and microbial genotypes and intra-microbiome interactions) and external (environmental) factors (Dastogeer et al., 2020; Hardoim et al., 2015; Lebeis, 2015; Neelakanta and Sultana, 2013). Recently, attention has been shifting toward the perspective of the existence of plants and microbes as a functioning ecological unit streamlining the postulate regarding them as a microbial consortium based on the holobiont concept (Cordovez et al., 2019). Advances in molecular techniques particularly amplicon metagenomics are rapidly allowing various culture-independent studies (Turner et al., 2013). For instance, in microbial ecology, it enables high throughput analysis of the diversity, structure, and composition of microbial communities, as well as the factors that influence the microbiome characteristics and their interactions with plants (Gilbert et al., 2014; Hilton et al., 2021; Turnbaugh et al., 2007; Turner et al., 2013). This development has tremendously improved the existing knowledge of the ecological functions of microbiomes (Cordovez et al., 2019).

The leaf is often associated with a relatively simple microbiome (Bodenhausen et al., 2013; Delmotte et al., 2009). The root microbiome is a rich ecosystem for soil-recruited microbial communities to thrive under the influence of certain environmental factors and characteristics of associated plants, including genotype and age which tend to shape the ecological diversity and composition (Hunter et al., 2014). The soil is the earth’s most complex habitat for microbes (Daniel, 2005). Its microbiota has been regarded as the secondary genome for plants due to its significant roles in agricultural production systems which include nutrient recycling, carbon sequestration, and formation of spectra of associations ranging from mutualistic, commensalistic, and pathogenic lifestyle (Berendsen et al., 2012; Newton et al., 2010). Attention is currently focused on exploring biotechnological approaches to engineer microbiomes of crop plants associated with important traits such as nutrient acquisition and disease suppression for sustainable crop health and yield security (Ryan et al., 2009).

Oil palm microbial community studies reported include culture-based isolation of rhizosphere bacteria and investigation of their growth-promoting potentials such as bacterial phosphate solubilization and inhibition of pathogens (Acevedo et al., 2014; Kirkman et al., 2022). The antagonistic potential of rhizosphere bacteria on the white rot fungus *Ganoderma boninense* associated with basal stem rot of oil palm has been reported (Nur Azura et al., 2016; Shariffah-Muzaimah et al., 2018). Other related studies include impacts of converting forest lands to oil palm plantations on the diversity of bacterial and fungal communities (Kaupper et al., 2020; Lee-Cruz et al., 2013; Tripathi et al., 2016) and the diversity of the bacterial community of oil palm which exhibits biodegradative effect on isoprene, a biogenic volatile organic compound (Carrión et al., 2020).

In the present study, we compared the fungal communities of leaf, root, and soil associated with asymptomatic and symptomatic oil palm trees naturally affected by leaf spot disease, using ITS2 rRNA gene metabarcoding. More specifically, our strategies for the current study were to determine whether (i) there are significant differences in the structure and composition of the mycobiomes in the same or different sites (ii) the structure and composition of the mycobiomes contribute significantly to the health status of the sampled trees (iii) there are significant differences in the network topological properties of the mycobiome of the asymptomatic and symptomatic samples.

## Materials and methods

### Study sites and sampling

**FIGURE 1:**
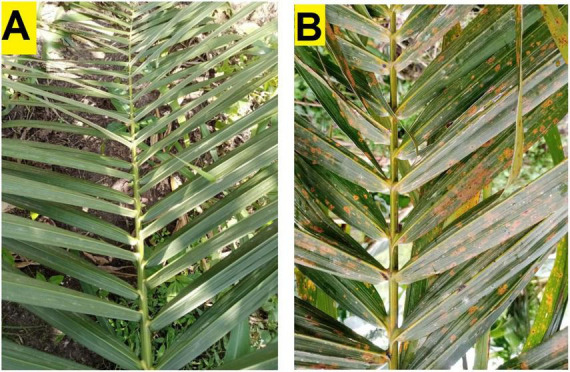
Oil palm fronds (leaves) from healthy asymptomatic tree **(A)** and symptomatic leaf-spot diseased tree **(B)**.

The fieldwork was carried out in November 2022 in five oil palm plots in the same ecological zone: two in Ore Town, Ondo State, and three in Okomu Village, Edo State, Nigeria. The oil palm farm in Ore town, Ondo state, is one of the research farms managed by the Rainforest Research Station (RFRS), an outstation of the Forestry Research Institute of Nigerian (FRIN) while the Edo state farm is private-owned and monitored by Nigerian Institute for Oil palm Research (NIFOR). The five oil palm plots were established in 2014 and adopted the same management practices, including the absence of fungicide or antibiotic application. Permission to collect plant specimens for the research was obtained from the Forestry Research Institute of Nigeria (FRIN) and the Nigerian Institute for Oil Palm Research (NIFOR). Sampling of leaves, roots and soils was conducted in plots established in 2014 in each of these farms in two locations: (i) 6°44′ 01.5″ N 4° 53′ 01.7″E and 6°44′ 41.5″ N 4° 52′ 21.3″E for Ore oil palm trees showing symptoms of leaf spot (considered as symptomatic trees), and those without symptoms of the disease (considered as asymptomatic trees), respectively, (ii) 6°40′ 17.12″ N 5° 49′ 21.7″E and 6°40′ 11.2″ N 5° 49′ 28.8″E for Okomu symptomatic trees and asymptomatic trees, respectively. Asymptomatic and symptomatic oil palm tree samples of leaf, root, and soil were collected in two replicates from Ore farm and three replicates from Okomu farm, resulting in a total of 5 biological replicates per treatment. Asymptomatic and symptomatic leaves (Figures 1A, B) were collected in well-labeled envelopes. Oil palm root and soil were sampled from a 20 cm hole dug 1 m away from each tree; approximately 10 g of roots and 200 g of soil samples were kept in zip-lock bags (Kirkman et al., 2022). A total of 30 samples (presented in Table 1) were collected. Formal identification of the plant specimens was done and the vouchers were deposited according to GPS coordinates and other sampling information. The samples were taken to the Forest Pathology Laboratory, Department of Forest Sciences, University of Helsinki, Viikki Campus Helsinki, Finland, and stored at −20°C before DNA isolation.

**TABLE 1:** Experimental samples and sampling information.

Sample ID	Replicate	Group	Source	Type	Location	GPS
Oleaf.4A	OLA	ORLA	Leaf	Asymptomatic	Okomu/Edo	6°40′ 11.2″ N 5° 49′ 28.8″E
OLeaf.5A	OLA	ORLA	Leaf	Asymptomatic	Okomu/Edo	6°40′ 11.2″ N 5° 49′ 28.8″E
OLeaf.6A	OLA	ORLA	Leaf	Asymptomatic	Okomu/Edo	6°40′ 11.2″ N 5° 49′ 28.8″E
OLeaf.1S	OLS	ORLS	Leaf	Symptomatic	Okomu/Edo	6°40′ 17.12″ N 5° 49′ 21.7″E
OLeaf.2S	OLS	ORLS	Leaf	Symptomatic	Okomu/Edo	6°40′ 17.12″ N 5° 49′ 21.7″E
OLeaf.3S	OLS	ORLS	Leaf	Symptomatic	Okomu/Edo	6°41′ 26.5″ N 5° 47′ 55.6″E
ORoot.4A	ORA	ORRA	Root	Asymptomatic	Okomu/Edo	6°40′ 11.2″ N 5° 49′ 28.8″E
ORoot.5A	ORA	ORRA	Root	Asymptomatic	Okomu/Edo	6°40′ 11.2″ N 5° 49′ 28.8″E
ORoot.6A	ORA	ORRA	Root	Asymptomatic	Okomu/Edo	6°40′ 11.2″ N 5° 49′ 28.8″E
ORoot.1S	ORS	ORRS	Root	Symptomatic	Okomu/Edo	6°40′ 17.12″ N 5° 49′ 21.7″E
ORoot.2S	ORS	ORRS	Root	Symptomatic	Okomu/Edo	6°40′ 17.12″ N 5° 49′ 21.7″E
ORoot.3S	ORS	ORRS	Root	Symptomatic	Okomu/Edo	6°40′ 17.12″ N 5° 49′ 21.7″E
OSoil.4A	OSA	ORSA	Soil	Asymptomatic	Okomu/Edo	6°40′ 11.2″ N 5° 49′ 28.8″E
OSoil.5A	OSA	ORSA	Soil	Asymptomatic	Okomu/Edo	6°40′ 11.2″ N 5° 49′ 28.8″E
OSoil.6A	OSA	ORSA	Soil	Asymptomatic	Okomu/Edo	6°40′ 11.2″ N 5° 49′ 28.8″E
OSoil.1S	OSS	ORSS	Soil	Symptomatic	Okomu/Edo	6°40′ 17.12″ N 5° 49′ 21.7″E
OSoil.2S	OSS	ORSS	Soil	Symptomatic	Okomu/Edo	6°40′ 17.12″ N 5° 49′ 21.7″E
OSoil.3S	OSS	ORSS	Soil	Symptomatic	Okomu/Edo	6°40′ 17.12″ N 5° 49′ 21.7″E
RLeaf.1A	RLA	ORLA	Leaf	Asymptomatic	Ore/Ondo	6°44′ 41.5″ N 4° 52′ 21.3″E
RLeaf.4A	RLA	ORLA	Leaf	Asymptomatic	Ore/Ondo	6°44′ 41.5″ N 4° 52′ 21.3″E
RLeaf.2S	RLS	ORLS	Leaf	Symptomatic	Ore/Ondo	6°44′ 01.5″ N 4° 53′ 01.7″E
RLeaf.5S	RLS	ORLS	Leaf	Symptomatic	Ore/Ondo	6°44′ 01.5″ N 4° 53′ 01.7″E
RRoot.1A	RRA	ORRA	Root	Asymptomatic	Ore/Ondo	6°44′ 41.5″ N 4° 52′ 21.3″E
RRoot.4A	RRA	ORRA	Root	Asymptomatic	Ore/Ondo	6°44′ 41.5″ N 4° 52′ 21.3″E
RRoot.2S	RRS	ORRS	Root	Symptomatic	Ore/Ondo	6°44′ 01.5″ N 4° 53′ 01.7″E
RRoot.5S	RRS	ORRS	Root	Symptomatic	Ore/Ondo	6°44′ 01.5″ N 4° 53′ 01.7″E
RSoil.1A	RSA	ORSA	Soil	Asymptomatic	Ore/Ondo	6°44′ 41.5″ N 4° 52′ 21.3″E
RSoil.2S	RSA	ORSS	Soil	Symptomatic	Ore/Ondo	6°44′ 01.5″ N 4° 53′ 01.7″E
RSoil.4A	RSA	ORSA	Soil	Asymptomatic	Ore/Ondo	6°44′ 41.5″ N 4° 52′ 21.3″E
Rsoil.5S	RSS	ORSS	Soil	Symptomatic	Ore/Ondo	6°44′ 01.5″ N 4° 53′ 01.7″E

OLA, Okomu asymptomatic leaf samples; OLS, Okomu symptomatic leaf samples; ORA, Okomu asymptomatic root samples; ORS, Okomu symptomatic root samples; OSA, Okomu asymptomatic soil samples; OSS, Okomu symptomatic soil samples; RLA, Ore asymptomatic leaf samples; RLS, Ore symptomatic leaf samples; RRA, Ore asymptomatic root samples; RRS, Ore symptomatic root samples; RSA, Ore asymptomatic soil samples; RSS, Ore symptomatic soil samples; ORLA, Okomu/Ore asymptomatic leaf samples; ORLS, Okomu/Ore symptomatic leaf samples; ORRA, Okomu/Ore asymptomatic root samples; ORRS, Okomu/Ore symptomatic root samples; ORSA, Okomu/Ore asymptomatic soil samples; ORSS, Okomu/Ore symptomatic soil samples.

### DNA extraction, amplification of ITS region, and sequencing

Before the DNA extraction, the oil palm leaf and root tissue samples were surface sterilized with 70% ethanol to eradicate surface contaminants (Saha et al., 2022). Subsequently, the leaf and root tissues were homogenized in liquid nitrogen using a sterilized mortar and pestle. Genomic DNA was isolated following a modified cetyl-trimethyl ammonium bromide (CTAB) procedure reported by Terhonen et al. (2011). DNA extraction from soil samples was performed using Qiagen DNeasy PowerSoil Pro Kit following the manufacturer’s recommendations with two additional repetitions of the washing steps. The concentration and purity of the resulting DNA samples were assessed by spectrophotometry, using NanoDrop ND-1000 (ThermoFisher Scientific, USA) and Qubit at Novogene. PCR amplification of the ITS2 region of the fungal metacommunity was performed at Novogene, United Kingdom. The two-primer pair, ITS3-2024F (GCATCGATGAAGAACGCAGC) and ITS4-2409R (TCCTCCGCTTATTGATATGC) designated as forward primer and reverse primer, respectively, were used in creating the amplicon sequence libraries (Toju et al., 2012). One leaf sample (OLeaf.1S) failed to amplify and was discarded, leaving 29 samples used for this study. The purification and sequencing of the PCR products of the 29 samples were conducted using the Illumina NovaSeq PE250 Platform. Across all the sequencing reads, approximately 250 nucleotides were obtained. All the obtained raw sequences are accessible in the Sequence Read Archive (SRA) of the National Center for Biotechnology Information (NCBI) under the BioProject ID PRJNA996782.

### Bioinformatics and statistical analyses

The pre-processing (demultiplexing, trimming of primer sequences, and removal of adapters) of the raw paired-end (PE) sequences was conducted at Novogene, UK. The obtained raw PE sequences were then analyzed using the QIIME2 pipeline (Bolyen et al., 2019) as previously described by Kim and Park (Kim and Park, 2021). DADA2 algorithm (Callahan et al., 2016) was employed in filtering low-quality reads, correcting errors, removing chimeric sequences, generating amplicon sequence variant (ASV) abundance tables (Kim and Park, 2021), and taxonomic assignments (Colgan and Burns, 2022). Samples were even rarefied to a minimum depth of 40,248 sequences. One sample (OR6) with the least counts (3,338 sequences) was removed. Amplicon sequence variants (ASVs) unique to a single sample or with frequencies < 4 sequences were also discarded. The representative sequences of the UNITE QIIME release for fungi (clustering at 97% similarity threshold, release 2022-11-29) generated from the Naïve Bayesian classifier were used to define fungal taxonomies based on the resulting ASVs from DADA2 (Nilsson et al., 2019; Rideout et al., 2014). Subsequently, ASVs remaining unclassified at kingdom and phylum levels were discarded (Addison et al., 2023).

Further downstream analysis and visualization were carried out in RStudio version 4.1.3 using the recommended R packages which include qiime2R (v0.99.6), phyloseq (v1.38.0), and tidyverse (v2.0.0).^1^ Representative samples were considered as the averaged taxonomic representatives of individual replicates obtained using the merged_samples function in phyloseq. Stacked bar plots showing the distinct and shared taxa within and among the sample types based on tree health status (sample type) at the level of phylum, and genus were generated using the comp barplot function in microViz R package version 0.11.0 (Barnett et al., 2021). The biomarker discovery algorithm linear discriminant analysis of effect size (LEfSe) was employed to identify the genera that accounted for the differences between sample types and groups. Bar plots were generated with differential relative abundance values of ASVs at the genus level (LDA scores > 3.5, and *p* < 0.05).

The estimates of Alpha-diversity indices (Observed Amplicon Sequence Variants (ASVs), Shannon, and Simpson) for the ASVs in each sample were calculated with the plot_richness function (from phyloseq). Mean probability (*p*-values) were determined with stat_compare means function (from ggpubr package v0.4.0). Box plots for the alpha-diversity indices were generated with ggplot2 package v3.4.2 (Wickham, 2016). A rarefaction curve (Supplementary Figure 1) was generated for the alpha-diversity analysis to confirm visually whether, at the selected sequencing depth diversity in each sample is sufficiently represented and additional sequencing effort may not have significantly yielded more taxa (Addison et al., 2023).

Beta-diversity was estimated for the different group categories based on the weighted-UniFrac distance for Principal Coordinate Analysis (PCoA) using ordinate/plot_ordination functions in PhyloSeq in R studio (v.4.1.3). Bray–Curtis dissimilarities was employed for Non-metric multidimensional scaling (NMDS) using vegan package for R. The graphs were visualized using ggplot2 (Wickham and Wickham, 2016). The statistical significance (set at *p* < 0.05) for alpha and beta diversity was determined by one-way analysis of variance (ANOVA) and non-parametric multivariate analysis, permutational multivariate analysis of variance (PERMANOVA) with permutation/pseudo-*F*-statistic test, respectively (Oksanen et al., 2007; Procter et al., 2022). Venn diagrams were generated to illustrate the distinct and shared ASVs using the trans_venn function in the microbial community ecology data analysis (microeco) R package (version 0.7.1) (Andrew et al., 2012) and the online interface of Bioinformatics & Evolutionary Genomics.^2^

Network analysis was performed using the file2meco function of the microeco R package (Liu et al., 2022) and visualized using Gephi software (ver.0.9.2). Taxonomic association networks were constructed from correlation data obtained from the relative abundance of the ASVs from the fungal communities (Wood et al., 2017). Network complexity was reduced with a Spearman’s correlation coefficient value (*r* ≥ 0.95) and statistically significant *P*-value < 0.001 (de Vries et al., 2018). Networks made up of nodes (representing ASVs) and edges (representing significant correlations between ASVs) were generated for each sample type and group. To characterize the network structure of each sample type and group, we evaluated six topological parameters, namely diameter, average path length, average weighted degree, average clustering coefficients, modularity, and connected components. Diameter measures the longest of shortest paths in a network while average path length is defined as the mean of the total number of steps along the shortest paths in all possible combination of node pairs in a network (Price et al., 2021). Average clustering coefficient describes the degree at which modules are present in a network (Deng et al., 2012). Modularity considers the number of sub-networks or modules which represent the compartments in a network (Newman, 2006). It also determines the extent of intra- or inter-modular connections within a network (Csardi and Nepusz, 2006). The weighted degree and closeness centrality were employed in identifying the potential keystone species (Gehlenborg et al., 2010; Jordán et al., 2007). The connectedness of a node within a network or sub-network is measured by the weighted degree (Espinoza et al., 2020) while the most central taxa determined by how close a node is to other nodes in the network is measured by closeness centrality (Ma’ayan, 2011).

## Results

### High-throughput Illumina NovaSeq PE250 sequencing information

A total of 3,435,417 high-quality sequences with an average of 118, 463 sequences, were obtained across the leaf, root, and soil samples of both asymptomatic (AS) and symptomatic diseased tree samples (SS). After denoising and quality filtering, the total number of reads per sample ranged between 3,338 and 151,463 for the samples collected from asymptomatic trees and 53, 859, and 154,327 for the symptomatic tree samples. Low Good’s coverage index (< 2.00%) obtained in the samples (including the excluded) indicates a very low proportion of singletons in the sequence reads. However, the inherent technical problem with PCR and sequencing accounted for the lowest reads (3,338) obtained in one (ORoot.6A) out of the 29 samples. This lowest read was discarded by rarefaction analysis and the next lowest read (40,248) was used as the sequencing depth. After filtering out unclassified ASVs that are present as singletons or assigned to plant or animal-specific sequences, a core set of 934,763 sequences allocated to the fungal domain was obtained from the remaining 28 samples with which further downstream analyses were conducted.

### Taxonomic distribution between sample types and across groups

**FIGURE 2:**
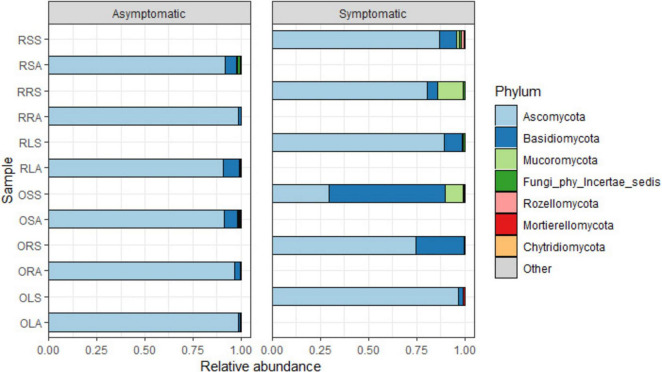
Relative abundance of fungal phyla from the annotated ASVs based on sample type. OLA, Okomu asymptomatic leaf samples; OLS, Okomu symptomatic leaf samples; ORA, Okomu asymptomatic root samples; ORS, Okomu symptomatic root samples; OSA, Okomu asymptomatic soil samples; OSS, Okomu symptomatic soil samples; RLA, Ore asymptomatic leaf samples; RLS, Ore symptomatic leaf samples; RRA, Ore asymptomatic root samples; RRS, Ore symptomatic root samples; RSA, Ore asymptomatic soil samples; RSS, Ore symptomatic soil samples.

**FIGURE 3:**
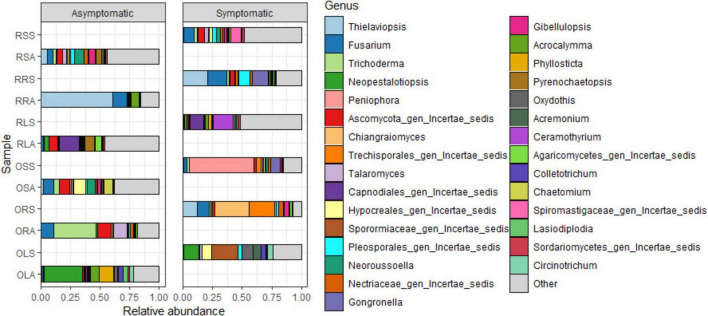
Relative abundance of fungal genera from the annotated ASVs based on sample type. OLA, Okomu asymptomatic leaf samples; OLS, Okomu symptomatic leaf samples; ORA, Okomu asymptomatic root samples; ORS, Okomu symptomatic root samples; OSA, Okomu asymptomatic soil samples; OSS, Okomu symptomatic soil samples. RLA, Ore asymptomatic leaf samples; RLS, Ore symptomatic leaf samples; RRA, Ore asymptomatic root samples; RRS, Ore symptomatic root samples; RSA, Ore asymptomatic soil samples; RSS, Ore symptomatic soil samples.

The quality-filtered and singleton-excluded sequences from the 28 samples (14 samples each from asymptomatic and symptomatic tree samples) were clustered into a total of 4,818 ASVs. The relative abundance of the phyla annotated from the ASVs found in AS and SS varied between sample types (Figure 2). Out of the 14 phyla identified, Ascomycota and Basidiomycota were the most dominant accounting for 94.2 and 4.7% of the total counts in AS, and 75 and 21.2% in SS, respectively. Next to these two major phyla is Mucoromycota with 0.44% relative abundance in AS represented in OLA, OSA, and RSA in AS, and 2.9% in SS which was actively represented in OSS, RRS and RSS in SS (Figure 2 and Supplementary Figure 2). The diversity of the sample types at the genus level (Figure 3 and Supplementary Tables 1A, B), showed that the relative abundances of most of the top 10 representatives shared by AS and SS are higher in the former. While *Neopestalotiopsis* is the most abundant genus for AS representing 8.0% of the identified amplicons compared to 2.0% in SS, *Peniophora* is the most abundant with 8.6% of the identified amplicons for SS compared to 0.1% in AS. The second most abundant genus for AS, *Fusarium*, accounted for 7.1% of the identified amplicons and 7.6% in SS while SS is an unassigned member of the order Trechisporales accounting for 8.5% of the identified amplicons but only 0.9% in AS. The relative abundance of each of *Thielaviopsis*, *Trichoderma*, and an unassigned member of Ascomycota lies around 6% in AS which is at least one-third higher than their respective values in SS. Together with *Neoroussoella*, *Phyllosticta*, and *Acrocalymma* which are unique to AS and *Chiangraiomyces*, *Ceramothyrium* and *Gongronella* are unique to SS, AS accumulates a lower relative abundance of the top 10 genera representing 47.2% of the identified amplicons in comparison with 51.3% found in SS.

**FIGURE 4:**
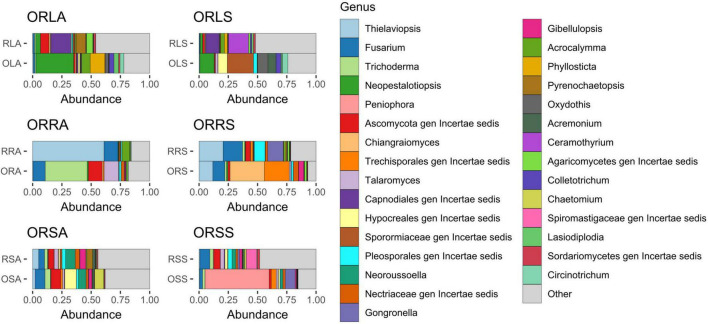
Relative abundance of fungal genera from annotated ASVs based on group. ORLA, Okomu/Ore asymptomatic leaf samples; ORLS, Okomu/Ore symptomatic leaf samples; ORRA, Okomu/Ore asymptomatic root samples; ORRS, Okomu/Ore symptomatic root samples; ORSA, Okomu/Ore asymptomatic soil samples; ORSS, Okomu/Ore symptomatic soil samples. OLA, Okomu asymptomatic leaf samples; OLS, Okomu symptomatic leaf samples; ORA, Okomu asymptomatic root samples; ORS, okomu symptomatic root samples; OSA, Okomu asymptomatic soil samples; OSS, Okomu symptomatic soil samples; RLA, Ore asymptomatic leaf samples; RLS, Ore symptomatic leaf samples; RRA, Ore asymptomatic root samples; RRS, Ore symptomatic root samples; RSA, Ore asymptomatic soil samples; RSS, Ore symptomatic soil samples.

Considering the genus diversity at the group level (Figure 4 and Supplementary Tables 1C–H), the topmost abundance values are acclaimed by different genera: *Neopestalotiopsis* (21% in ORLA), an unassigned member of the family Sporormiaceae (19.0% in ORLS), *Thielaviopsis* (17.7% in ORRA), an unassigned member of the order Trechisporales (16.9% in ORRS), *Neoroussoella* (7.8% in ORSA) and *Peniophora* (24.0% in ORSS). Notably, the abundance of *Neopestalotiopsis* in ORLA is about 4 times that of ORLS. The presence of *Phylosticta* and *Acrocalymma* in ORLA and *Ceramothyrium* in ORLS at a higher level reflects the observations of the genus diversity based on sample type. The relative abundance of *Colletotrichum* and *Lasiodiplodia* is much higher in ORLA compared to ORLS. *Pseudopestalotiopsis* and *Cylindroaseptospora* with 2.5–3.5% relative abundance that is exclusive to ORLA, and *Trichomerium*, *Oxydothis*, *Ruinenia*, and *Acremonium* with 2.4–3.4% relative abundance that is exclusive to ORLS, and together with other representatives, the top 10 genera accounts for *ca*. 59% of the identified amplicons found in each of ORLA and ORLS. The total relative abundance of the top 10 genera found in ORRA (75.0%) and ORRS (76.2%) is higher than in other groups. *Fusarium* accumulated at a higher level in ORRA (14.1%) and ORRS (14.8%) compared to other groups. *Trichoderma* is present at a much higher abundance (*ca*. 15%) in ORRA as much as *Chiangraiomyces* is in ORRS compared to other groups. *Leptobacillium* (2.5%) is exclusive to ORRS. *Gongronella* presents a relative abundance of 2.9 and 4.6% in ORRS and ORSS, respectively. Apart from ORRA where the relative abundance of *Acrocalymma* is about two-thirds of its highest value found in ORLA, the presence of the genus is much lower (≤ 1%) in other groups. Generally, all the top 10 genera found in both ORSA and ORSS have higher relative abundance in the former; *Chaetomium* with a relative abundance of 3.7% is exclusive to ORSA, and *Aspergillus* (2.08%) is exclusive to ORSS. ORSA shared the genus *Talaromyces*, *Pyrenochaetopsis*, and *Gibellulopsis* with ORRA, ORLA, and ORRS, respectively, and their relative abundance ranged between 2.4 and 5.3%. Moreover, the top 10 genera found in ORSA and ORSA including the unassigned taxa accounted for 47.3 and 58.7% of the identified amplicons, respectively.

**FIGURE 5:**
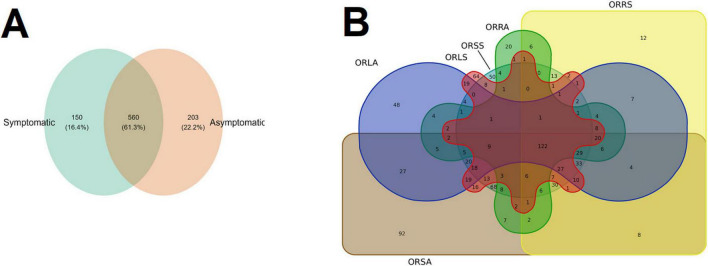
Venn diagrams showing the shared and unique number of genus **(A)** between the sample types **(B)** across the group. ORLA, Okomu/Ore asymptomatic leaf samples; ORLS, Okomu/Ore symptomatic leaf samples; ORRA, Okomu/Ore asymptomatic Root samples; ORRS, Okomu/Ore symptomatic root samples; ORSA, Okomu/Ore asymptomatic soil samples; ORSS, Okomu/Ore symptomatic soil samples.

For further analyses of the diversity of the fungal community, we focused on the shared and unique genera in the sample type and group using Venn diagrams (Figures 5A, B). At the level of genus, we detected 913 genera. According to sample type, 203 genera (22.2%) are unique to AS, 150 (16.4%) to SS while 560 (61.3%) are shared by both AS and SS (Figure 5A). Focusing on the groups, the most shared number of genera (122) was found for all the six groups, and those found in different group combinations ranged between 1 and 68 (shared by ORSA and ORSS), and the exclusive orders found in ORLA (48), ORLS (64), ORRA (20), ORSA (92), and ORSS (50) (Figure 5B).

### Taxa enrichment of the group based on linear discriminant analysis effect size (LEfSe)

**FIGURE 6:**
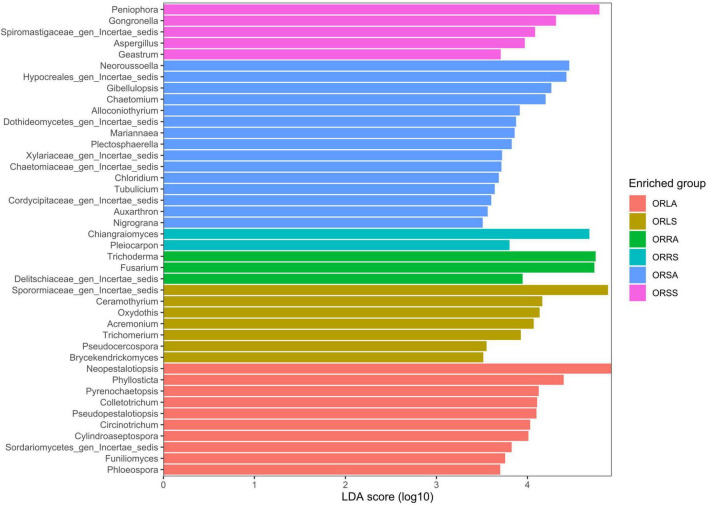
LEfSe bar plot based on significantly enriched genera across groups. ORLA, Okomu/Ore asymptomatic leaf samples; ORLS, Okomu/Ore symptomatic leaf samples; ORRA, Okomu/Ore asymptomatic root samples; ORRS, Okomu/Ore symptomatic root samples; ORSA, Okomu/Ore asymptomatic soil samples; ORSS, Okomu/Ore symptomatic soil samples.

The taxa enrichment level of the groups followed the order: ORSA > ORLA > ORLS > ORSS > ORRA > ORRS; with the highest (15) and lowest (2) number of significantly enriched genera found in ORSA and ORRS, respectively. *Neopestalotiopsis* (in ORLA), an assigned member of the family Sporormiaceae (in ORLS), *Trichoderma* (in ORRA), *Chiangraiomyces* (in ORRS), *Neoroussoella* (in ORSA), and *Peniophora* (in ORSS) dominated the groups (Figure 6).

### Mycobiome structure and composition

**FIGURE 7:**
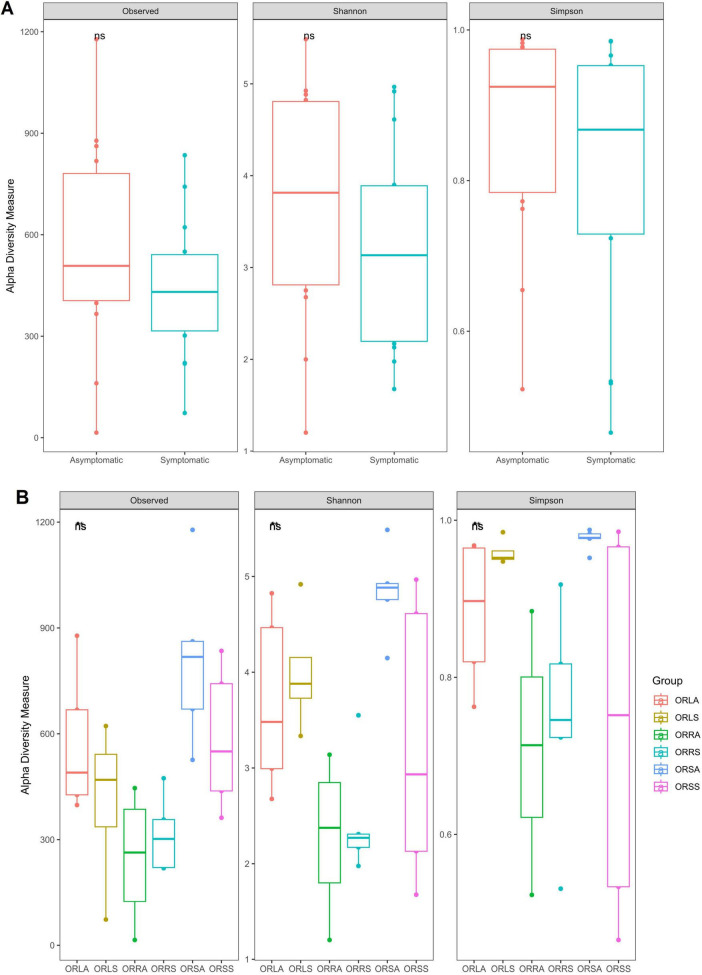
Alpha diversity of the fungal communities based on **(A)** sample type and **(B)** group. ORLA, Okomu/Ore asymptomatic leaf samples; ORLS, Okomu/Ore symptomatic leaf samples; ORRA, Okomu/Ore asymptomatic root samples; ORRS, Okomu/Ore symptomatic root samples; ORSA, Okomu/Ore asymptomatic soil samples; ORSS, Okomu/Ore symptomatic soil samples.

The rarefaction curve showed that all samples attained asymptotes (Supplementary Figure 1). Approximately 40,248 sequence reads from rarefaction analysis were utilized across the remaining 28 samples to obtain a meaningful richness estimate. Species richness and evenness of the AS and SS fungal communities evaluated by the diversity indices (number of observed species, Shannon and Simpson index) indicated that the intra-variability in richness and evenness is higher in AS samples compared to SS samples. However, the difference is not significant (*p* > 0.05) in the SS community (Figure 7A). At the group level, only ORSA exhibited significantly higher richness than ORLA (*p* = 0.016), ORLS (*p* = 0.032), ORRA (*p* = 0.016) and ORRS (*p* = 0.008). The differences found for other group combinations including ORSA/ORSS are not statistically significant (*p* > 0.05) (Figure 7B). Similarly, further comparison of the composition of the communities based on principal coordinate analysis (PCoA) using Bray-Curtis dissimilarity and PERMANOVA revealed a non-significant difference between AS and SS samples based on sample type (*F* = 0.34, *R*^2^ = 0.04, *p* = 0.60) and location (*F* = 0.02, *R*^2^ = 0.05, *p* = 0.89). As illustrated in the NMDS plots, PCoA Axis 1 accounted for 12.4% of the variation observed in the fungal communities of the samples. There was no definite clustering of fungal communities according to location (Supplementary Figure 4a) and sample type (Supplementary Figure 4b) in individual samples.

### Network structure of the fungal communities

**FIGURE 8:**
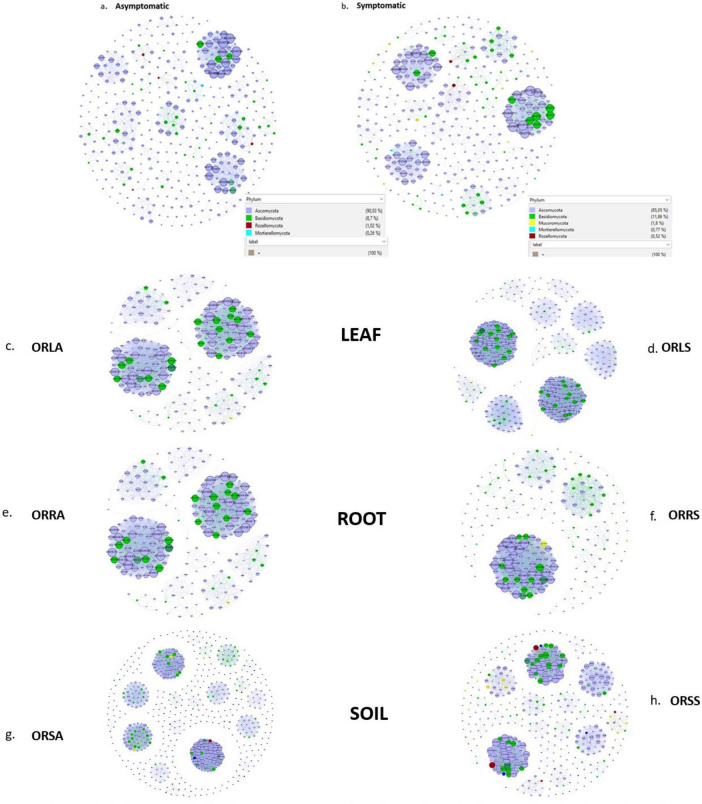
Association network maps of oil palm tissue and soil fungal communities generated according to relative abundance correlation data for sample type: **(a)** asymptomatic **(b)** symptomatic, and group: **(c)** ORLA **(d)** ORLS **(e)** ORRA **(f)** ORRS **(g)** ORSA **(h)** ORSS. ORLA, Okomu/Ore asymptomatic leaf samples; ORLS, Okomu/Ore symptomatic leaf samples; ORRA, Okomu/Ore asymptomatic root samples; ORRS, Okomu/Ore symptomatic root samples; ORSA, Okomu/Ore asymptomatic soil samples; ORSS, Okomu/Ore symptomatic soil samples.

The AS microbiome network contains 402 taxa as nodes with 1,697 interactions as edges while the SS network has 393 nodes and 2,555 edges (Figures 8a, b). The topological parameters evaluated for each network structure is presented in Table 2. A higher number (56) of connected components was found in AS compared to 47 in SS in contrast to their average clustering values (0.32 in AS/ 0.37 in SS) and modularity values (0.85 in AS/ 0.88 in SS). The average path length (APL) and diameter were comparatively higher in AS compared to SS. In the group networks (Figures 8c–h), the highest (664) and the lowest (237) number of nodes were found in ORSA and ORRA, respectively, while the highest (12,512) and the lowest (3,005) for edges were found in ORLS and ORLA, respectively. The modularity values ranged between 0.38 (in ORRS) and 0.80 (ORSS) and are largely group dependent. Apart from the root samples, all AS and SS samples in each group had close modularity values. The APL and diameter had the same value in the groups. The potential key stone taxa were identified in the AS (8 taxa) and SS (18 taxa) samples based on weighted degree (weighted in-/out-degree (> 10)) and closeness centrality (1) scores (Table 3). Although, these indices could not be applied at the group level as there were no clear-cut differences between many taxa.

**TABLE 2:** Network topological parameters for sample types and groups.

Network topological parameters	Asymptomatic	Symptomatic	ORLA	ORLS	ORRA	ORRS	ORSA	ORSS
Network diameter	9.0	7.0	1.0	1.0	1.0	1.0	1.0	1.0
Average path length	2.2	1.8	1.0	1.0	1.0	1.0	1.0	1.0
Average weighted degree	4.19	6.47	7.91	30.44	17.38	13.12	14.15	10.54
Average clustering coefficient	0.32	0.37	0.44	0.5	0.49	0.44	0.47	0.46
Modularity	0.85	0.87	0.60	0.67	0.66	0.38	0.76	0.80
Connected components	56	47	74	16	14	39	91	58

Sample types: Asymptomatic, symptomatic; Groups: ORLA, Okomu/Ore asymptomatic leaf samples; ORLS, Okomu/Ore symptomatic leaf samples; ORRA, Okomu/Ore asymptomatic Root samples; ORRS, Okomu/Ore symptomatic root samples; ORSA, Okomu/Ore asymptomatic soil samples; ORSS, Okomu/Ore symptomatic soil samples.

**TABLE 3:** Potential key stone species in the asymptomatic and symptomatic (leaf-spot diseased) oil palm trees.

S/N	Taxa	Phylum	Order	Species	Relative abundance	Weighted in-degree	Weighted-out degree	Weighted degree	Closeness centrality
**Asymptomatic**									
1	ASV1171	Ascomycota	Onygenales	*Currahomyces indicus*	0.01	13	17	30	1
2	ASV1667	Ascomycota	Chaetothyriales	*Chaetothyriales* sp.	0.04	14	16	30	1
3	ASV2099	Ascomycota	Helotiales	*Kendrickiella phycomyces*	0.03	15	15	30	1
4	ASV2713	Basidiomycota	Agaricostilbales	*Ruinenia pyrrosiae*	0.02	16	14	30	1
5	ASV2828	Basidiomycota	Agaricales	*Stropharia* sp.	0.01	17	13	30	1
6	ASV2963	Basidiomycota	Agaricales	*Xanthagaricus necopinatus*	0.01	18	12	30	1
7	ASV3934	Ascomycota	Pleosporales	*Byssosphaeria* sp.	0.04	19	11	30	1
8	ASV4003	Ascomycota		Ascomycota sp.	0.01	20	10	30	1
**Symptomatic**									
1	ASV1934	Ascomycota	Capnodiales	*Eriosporella bambusicola*	0.02	22	15	37	1
2	ASV1916	Ascomycota	Mycosphaerellales	*Teratosphaeria* sp.	0.07	21	16	37	1
3	ASV1908	Ascomycota	Capnodiales	*Capnodiales* sp.	0.02	20	17	37	1
4	ASV1877	Ascomycota	Capnodiales	*Capnodiales* sp.	0.01	19	18	37	1
5	ASV1876	Ascomycota	Capnodiales	*Capnodiales* sp.	0.02	18	19	37	1
6	ASV1839	Ascomycota	Capnodiales	*Capnodiales* sp.	0.03	17	20	37	1
7	ASV1804	Ascomycota	Capnodiales	*Capnodiales* sp.	0.01	16	21	37	1
8	ASV1750	Ascomycota	Lecanorales	*Bacidia neosquamulosa*	0.01	14	23	37	1
9	ASV1754	Ascomycota	Lecanorales	*Bacidia neosquamulosa*	0.02	15	22	37	1
10	ASV1671	Ascomycota	Chaetothyriales	*Brycekendrickomyces acaciae*	0.06	13	24	37	1
11	ASV1640	Ascomycota	Chaetothyriales	*Cyphellophora* sp.	0.03	12	25	37	1
12	ASV1632	Ascomycota	Chaetothyriales	*Cyphellophora livistonae*	0.03	11	26	37	1
13	ASV1561	Ascomycota	Phaeomoniellales	*Celotheliaceae* sp.	0.02	10	27	37	1
14	ASV2716	Basidiomycota	Agaricostilbales	*Ruinenia pyrrosiae*	0.03	27	10	37	1
15	ASV2715	Basidiomycota	Agaricostilbales	*Ruinenia pyrrosiae*	0.05	26	11	37	1
16	ASV2714	Basidiomycota	Agaricostilbales	*Ruinenia pyrrosiae*	0.05	25	12	37	1
17	ASV2638	Ascomycota	Helotiales	*Helotiales* sp.	0.02	24	13	37	1
18	ASV2269	Ascomycota	Diaporthales	*Chiangraiomyces bauhiniae*	0.03	23	14	37	1

## Discussion

One of the unexpected findings of this study was the presence of ASVs identified as potential pathogens (e.g., *Thielaviopsis*, *Neopestalotiopsis*, *Pseudopestalotiopsis*, *Colletotrichum*) in apparently healthy trees (Figure 1A). Based on this observation, the sampled oil palm trees were classified as “symptomatic” (with leaf spot) and “asymptomatic” (without leaf spot).

Additionally, many previous studies on oil palm microbiome utilized culture-based approaches (Kirkman et al., 2022). Consequently, only scanty information is available on the microbiome composition and structure of oil palm. In the present study, the consideration of two sampling sites of close geographical proximity could account for the lack of statistically significant differences in the diversity of fungal biota of the leaf, root, and soil of asymptomatic and symptomatic leaf spot diseased oil palm trees from the two agricultural ecosystems. However, further analysis, treating asymptomatic and symptomatic samples from the same tissue or soil type as a single group (without considering their source location), accounted for most of the findings in this study. These results are congruent with the earlier reports on the diversity of the fungal community of root, rhizosphere, and soil of oil palm (Kirkman et al., 2022) and the dynamics of soil bacterial and fungal biota under the influence of secondary succession (Lin et al., 2021).

Earlier studies documented Ascomycota and Basidiomycota as the two phyla associated with oil palm affected by fatal yellowing disease (FYD) (de Assis Costa et al., 2018). Similarly, in this study, Ascomycota and Basidiomycota which also constitute the dominant phyla in all the samples along with other phyla with relatively low abundance were discovered. This observation therefore suggests the duo as the dominant phyla associated with oil palm fungal communities. Similar findings have also been reported on the characterization of endophytic mycobiota in palms (Guo et al., 2001), the oak tree *Quercus robur* (Gonthier et al., 2006), and the medicinal plant *Achyranthes aspera* (John and Mathew, 2017). Additionally, most of the Basidiomycetous fungi identified in this work have been reported as endophytes from various studies including the first report of Basidiomycetous endophytes from oil palm by Rungjindamai et al. (2008). The higher abundance of Basidiomycota observed in symptomatic samples relative to asymptomatic samples might be attributed to the successional changes occurring after colonization of oil palm tissue and soil, which contribute to the manifestation of the observed leaf spot symptoms. Hypocreales and Pleosporales represent the dominant orders in both AS and SS. Hypocreales were enriched in the root and soil samples except the soil-symptomatic samples. This order has also been reported to be predominant in root in a similar study on ecological diversity of oil palm that compared root, rhizosphere, and soil microbiome (Kirkman et al., 2022) and the effect of selective-logging on activities of oil palm fungal communities (Kerfahi et al., 2014). Pleosporales exhibiting high abundance in the leaf samples reinforces the postulate that the order is grossly associated with oil palm (Wong et al., 2021). Members of the Pleosporales are known to exhibit diverse ecological niches and have been identified as epiphytes, endophytes, saprotrophs, pathogens, and parasites of fungi and insects from various studies (Kruys and Wedin, 2009; Ramesh, 2003). This further substantiates the persistence of the order across all the investigated samples. The predominance of the order Russulales which accommodates the ectomycorrhizal genera in the symptomatic soil samples with little or no impact in restricting the progression of the disease cannot be explained.

### Occurrence of leaf spot-associated genera and taxa signatures across the samples’ categories

Many of the genera previously documented as potential causative agents of leaf spot disease in crops and trees were found in both asymptomatic and diseased trees. These include *Neopestalotiopsis*, *Pseudopestalotiopsis*, *Colletotrichum*, *Lasiodiplodia*, *Ceramothyrium*, and two others that were unidentified beyond the order Capnodiales among the top 30 ASVs. Surprisingly, all the genera found in common to both asymptomatic and symptomatic samples exhibit higher abundance in the former. The only exception is *Ceramothyrium* (Chaetothyriales) whose abundance is much higher in the SS samples and has the highest abundance in the symptomatic leaf samples. *Neopestalotiopsis* and *Pseudopestalotiopsis* have previously been associated with oil palm leaf spot disease in Brazil (Suwannarach et al., 2013) and Malaysia (Ismail et al., 2017) and Indonesia (Yurnaliza et al., 2021); the genera were also surprisingly predominant in the asymptomatic leaves of oil palm in this study. Meanwhile, the conclusion from another work on FYD stated that the two genera and *Pestalotiopsis* found in lower abundance in this study may not be directly associated with the disease as they are readily isolated from oil palm plantations where foliar lesions are observed (de Assis Costa et al., 2018). Moreover, another report also enlisted *Colletotrichum* sp. among the endophytic fungi associated with the asymptomatic medicinal plant *Achyranthes aspera* (John and Mathew, 2017). It is possible that some of the identified beneficial endophytes including *Colletotrichum* sp. and *Trichoderma asperellum* with higher abundance in asymptomatic samples have contributed to restricting the emergence of pathogenicity factors of these fungi (Boari, 2008; John and Mathew, 2017). In contrast, the most dominant genus found in symptomatic leaves is an unassigned member of the family Sporormiaceae (Pleosporales). Previous reports by de Assis Costa et al. (2018) had associated *Colletotrichum*, *Pestalotiopsis*, and *Fusarium* with FYD based on the findings from their study. However, earlier reports had already shown that the disease was not reproducible in an inoculation experiment conducted using the trio (Boari et al., 2012; Boari, 2008). Since these genera were also found but in lower abundance in SS in this study, they might be among the opportunistic pathogens inhabiting the tissue and soil awaiting a favorable condition to cause or contribute to the severity of foliar disease of oil palm. Together with other yet-to-be-identified genera discovered in this study, especially in the symptomatic samples, so many putative causal agents of oil palm foliar diseases including leaf spot might still be unknown. These causal agents may include two or more pathogens which suggest the disease might likely be due to the activity of a consortia of pathogens or pathobiome.

Asymptomatic soil samples were enriched with more fungal taxa compared to other groups which is in contrast with the report made by Darriaut et al. (2023) on the investigation of fungal biota associated with symptomatic and asymptomatic soils of grapevine. Furthermore, the asymptomatic soil samples exhibit the highest diversity comprising soil-borne saprobes such as *Neoroussoella* and *Gibellulopsis* (Hirooka et al., 2014) as found in other works which further supports the claim that, they are a reservoir of phytotaxa that are attracted to the rhizosphere and subsequently selected by the roots (Byers et al., 2020; Darriaut et al., 2023). The symptomatic soil samples are predominantly enriched in saprobic genera including *Peniophora* (Roozbeh et al., 2013), *Gongronella* (Zhang et al., 2019), and *Aspergillus* (Nji et al., 2023). The root samples are the least enriched with the asymptomatic samples having the lowest taxa at the order level and the symptomatic at the genus level. The observation of a higher abundance of putative disease-suppressing genus *Trichoderma* (*T. asperellum*) in the asymptomatic root could explain its tolerance against leaf spot disease. In addition to the genera described above for asymptomatic leaves, other economically important genera discovered with the biomarker discovery algorithm LEfSe include *Phyllosticta* which species have been reported to cause oil palm leaf spot in Malaysia (Nasehi et al., 2020). Among the fully assigned taxa signatures in the symptomatic leaves are *Oxydothis* which is associated with leaf spots in palms (Fröhlich and Hyde, 1994), *Pseudocercospora* which causes *Cercospora* leaf spot, a notorious foliar disease in olives (Ávila et al., 2005). Others include the foliar epiphytes genera *Ceramothyrium* and *Trichomerium* which have also been ascribed to leaf spot in many plants (Chomnunti et al., 2012; Hongsanan et al., 2015).

### Alpha and beta diversity analyses

There was no statistically significant difference in the diversity and richness of AS and SS in both sample types and location. Furthermore, the diversity metrics were generally low across the samples but higher in AS. This finding reinforces the report by Wang and Foster (2015) that lower diversity is more probable in plantations established in former agricultural ecosystems. Also, this observation mirrors our finding with the PCoA analysis of the beta diversity of the samples based on location and sample type as there was no definite clustering pattern among the individual samples of AS and SS investigated (see Supplementary file). Meanwhile, the earlier study by de Assis Costa et al. (2018) reported definite clustering of FYD-asymptomatic samples and scattering of FYD-symptomatic samples across the 2-dimensional space of principal component analysis (PCA) graph.

### Association networks and the potential keystone species

Correlation-based taxonomic networks are widely used for depicting microbiome associations because they can easily be calculated, scaled, subjected to a plethora of asymptotic statistical inferences, and employed to differentiate inverse connections (Song et al., 2012). The network analyses performed in this study partly supports our previous finding that there is no significant difference in microbial diversity in most of the categorizations (soil, leaf, root) considered for the samples. Additionally, some taxa which were previously unnoticed (especially the rare taxa) in the previous set of analyses as important constituent of the microbial community were unraveled as potential keystone species (Wood et al., 2017) using the network metrics described above. Such species in the group association networks play a pivotal role in ecosystem functioning (Gehlenborg et al., 2010), and their extinction may affect the stability of the communities (Berry and Widder, 2014). All the group networks except the symptomatic root samples are characterized with high modularity. This indicates coherent node connections within the modules and sparse connections between modules. Therefore, the modules (communities) largely accommodate balanced and similar ecological units with minimal environmental disturbance and species loss. On the other hand, the comparatively lower modularity index found in the symptomatic root samples suggests that within this network, many taxa are more frequently associated with other categories of taxa than they are connected to each other (Burgos et al., 2007; Ding et al., 2015). Since environmental variability has been reported to exert a positive influence on modularity (Kreimer et al., 2008), the close modularity values observed in the two microbial communities further points to their geographical proximity as agroecosystems.

## Conclusion

This is the first report comparing the structure and composition of the fungal community of asymptomatic and symptomatic leaf-spot diseased oil palm trees sampled from the tropics in Nigeria. Apart from the arrays of putative oil palm leaf spot pathogens, other categories of fungi in the asymptomatic samples especially the leaf observed in this study should be further investigated to unravel the actual causal agents of oil palm leaf spot disease. In addition, *Trichoderma asperellum* occurring in higher abundance in the asymptomatic root samples could be responsible for the pathogen suppression displayed by the asymptomatic trees against the disease. Hence, members of the genus could be explored as biocontrol agents against oil palm leaf spot disease. The findings from the association network study also support our observation that there is no significant difference between the asymptomatic and leaf-spot-diseased oil palm trees in the diversity analyses. Our observation of the rare taxa as a large proportion of the keystone species suggests further investigation of this important constituent of the fungal microbial communities. This is critical to identify their potential ecosystem functions and how they might be linked to other members of the communities.

## Data Availability

The datasets presented in this study can be found in online repositories. The names of the repository/repositories and accession number(s) can be found in this article/Supplementary material.

## Footnotes

1
https://github.com/jbisanz/qiime2r?search=1

2
https://github.com/uw-madison-microbiome-hub/Microbiome_analysis_in-_R/blob/master/readme.md
