# Supplementary material for: Mycobiome analysis of leaf, root, and soil of symptomatic oil palm trees (Elaeis guineensis Jacq.) affected by leaf spot disease
Source: Front Microbiol. 2024 Dec 6;15:1422360. doi: 10.3389/fmicb.2024.1422360 (PMC11659247; doi:10.3389/fmicb.2024.1422360)
Supplement: Supplementary file 1 [file Data_Sheet_1.pdf]

Supplementary files

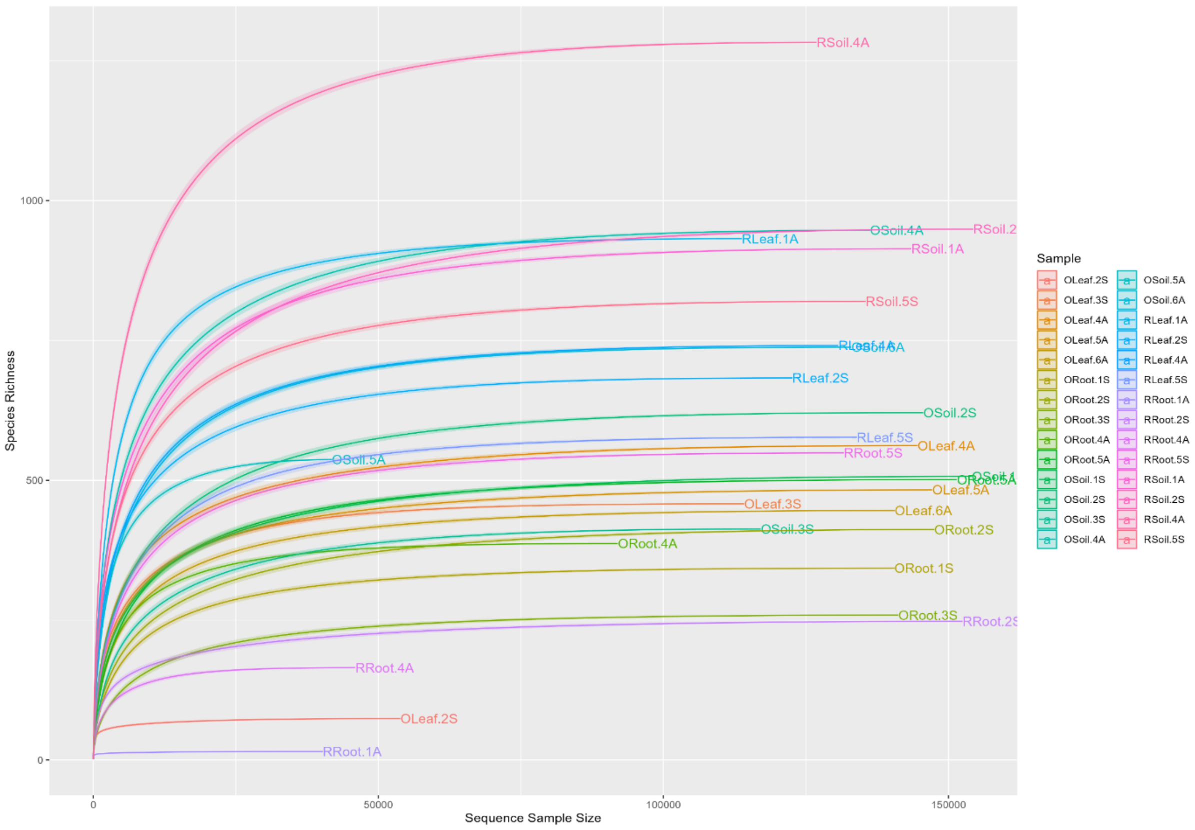

Fig. S1: Rarefaction curve for alpha-diversity analysis indicating a sufficient representation of each sample at the selected depth.

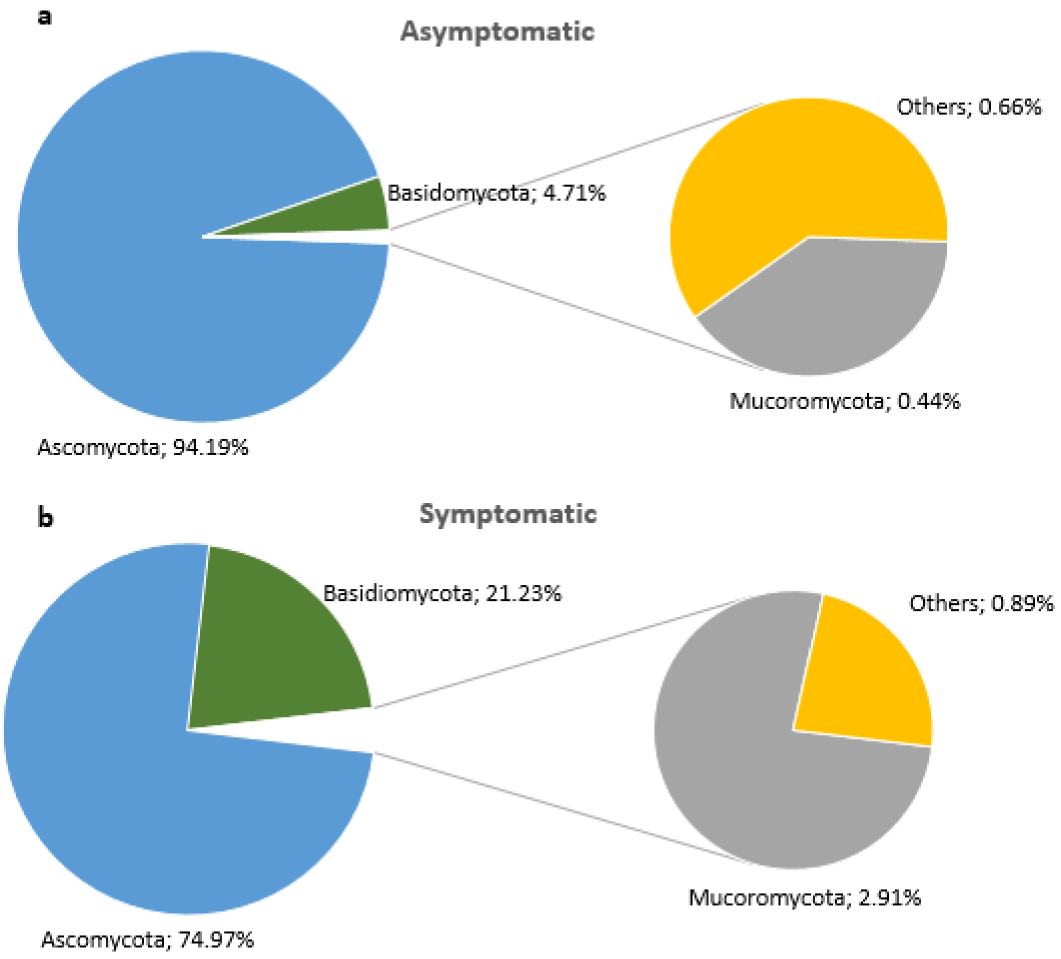

Fig. S2: Relative abundance of the dominant phyla based on sample type.

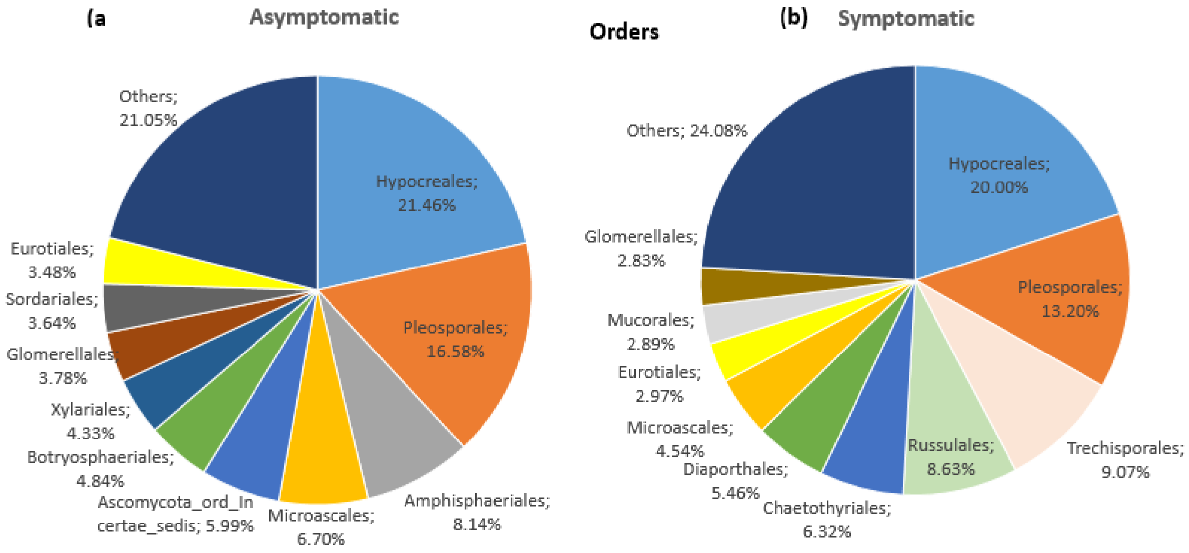

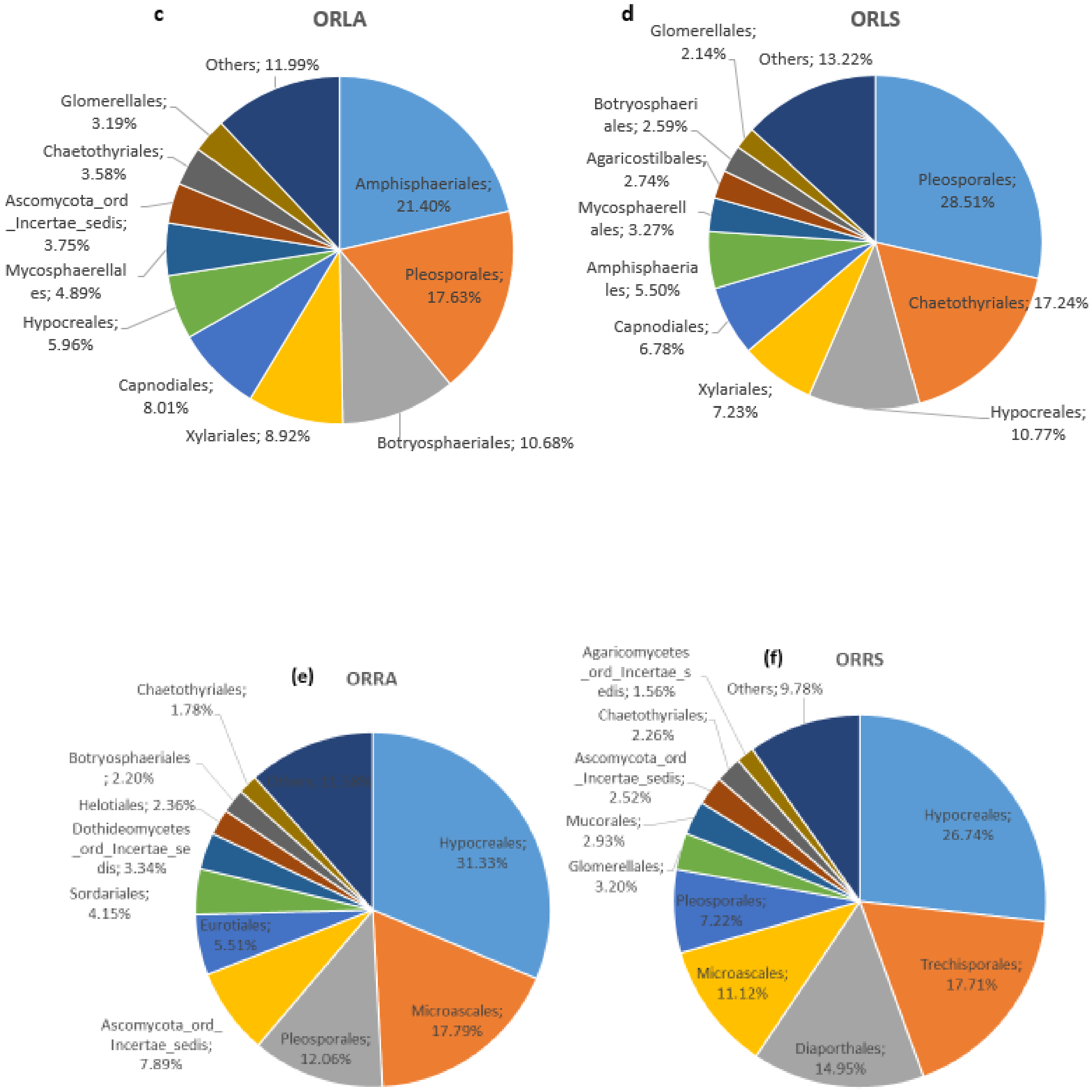
c
ORLA
Others; 11.99%
Glomerellales; 3.19%
Chaetothyriales; 3.58%
Ascomycota_ord _Incertae_sedis; 3.75%
Mycosphaerellales; 4.89%
Hypocreales; 5.96%
Capnodiales; 8.01%
Xylariales; 8.92%
Botryosphaeriales; 10.68%
Pleosporales; 17.63%
Amphisphaeriales; 21.40%
d
ORLS
Glomerellales; 2.14%
Others; 13.22%
Botryosphaeriales; 2.59%
Agaricostilbales; 2.74%
Mycosphaerellales; 3.27%
Amphisphaeriales; 5.50%
Capnodiales; 6.78%
Xylariales; 7.23%
Hypocreales; 10.77%
Chaetothyriales; 17.24%
Pleosporales; 28.51%
(e)
ORRA
Chaetothyriales; 1.78%
Botryosphaeriales ; 2.20%
Helotiales; 2.36%
Dothideomycetes _ord_Incertae_sedis; 3.34%
Sordariales; 4.15%
Eurotiales; 5.51%
Ascomycota_ord_ Incertae_sedis; 7.89%
Pleosporales; 12.06%
Microascales; 17.79%
Hypocreales; 31.33%
(f)
ORRS
Agaricomycetes _ord_Incertae_sedis; 1.56%
Others; 9.78%
Chaetothyriales; 2.26%
Ascomycota_ord _Incertae_sedis; 2.52%
Mucorales; 2.93%
Glomerellales; 3.20%
Pleosporales; 7.22%
Microascales; 11.12%
Diaporthales; 14.95%
Trechisporales; 17.71%
Hypocreales; 26.74%

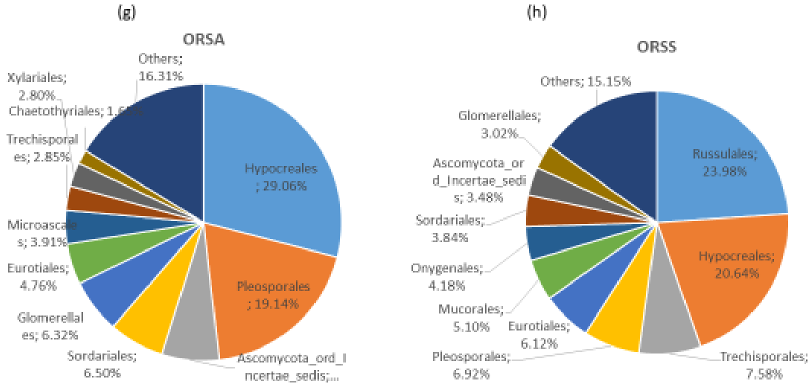

Fig. S3: Relative abundance of fungal orders from annotated ASVs based on sample type (a &b) and group (c-h).

Table S1A-H: Relative abundance of the top 10 fungal genera from annotated ASVs in the sample types and groups.

A: **Asymptomatic**

Genus	Rel. abund. (%)
Neopestalotiopsis	8.01
Fusarium	7.06
Thielaviopsis	6.39
Trichoderma	6.01
Ascomycota_gen_Incertae_sedis	5.99
Capnodiales_gen_Incertae_sedis	2.91
Neoroussoella	2.87
Phyllosticta	2.84
Hypocreales_gen_Incertae_sedis	2.58
Acrocalymma	8.01
Others	47.33

B: **Symptomatic**

Genus	Rel. abund. (%)
Peniophora	8.61
Trechisporales_gen_Incertae_sedis	8.54
Fusarium	7.60
Sporormiaceae_gen_Incertae_sedis	5.50
Chiangraiomyces	5.40

Thielaviopsis	4.12
Pleosporales_gen_Incertae_sedis	3.27
Ceramothyrium	3.15
Gongronella	2.69
Ascomycota_gen_Incertae_sedis	2.43
Others	48.69

C: ORLA

Genus	Rel. abund. (%)
Neopestalotiopsis	21.04
Phyllosticta	7.60
Capnodiales_gen_Incertae_sedis	7.28
Acrocalymma	4.95
Ascomycota_gen_Incertae_sedis	3.75
Pyrenochaetopsis	3.47
Pseudopestalotiopsis	3.21
Cylindroaseptospora	2.70
Colletotrichum	2.57
Lasiodiplodia	2.53
Others	40.90

D: **ORLS**

Genus	Rel. abund. (%)
Sporormiaceae_gen_Incertae_sedis	18.96
Ceramothyrium	10.93
Capnodiales_gen_Incertae_sedis	5.78
Neopestalotiopsis	5.39
Trichomerium	3.42
Oxydothis	3.38
Hypocreales_gen_Incertae_sedis	2.89
Ruinenia	2.74
Pleosporales_gen_Incertae_sedis	2.69
Acremonium	2.38
Others	41.44

E: **ORRA**

Genus	Rel. abund. (%)
Thielaviopsis	17.73
Trichoderma	14.93

Fusarium	14.09
Ascomycota_gen_Incertae_sedis	7.89
Talaromyces	5.28
Delitschiaceae_gen_Incertae_sedis	4.40
Dothideomycetes_gen_Incertae_sedis	3.34
Pleosporales_gen_Incertae_sedis	2.71
Sordariales_gen_Incertae_sedis	2.52
Acrocalymma	2.15
Others	24.96

F**: ORRS**

Genus	Rel. abund. (%)
Trechisporales_gen_Incertae_sedis	16.90
Chiangraiomyces	14.87
Fusarium	14.82
Thielaviopsis	10.61
Pleosporales_gen_Incertae_sedis	5.33
Nectriaceae_gen_Incertae_sedis	3.10
Gongronella	2.91
Gibellulopsis	2.70
Ascomycota_gen_Incertae_sedis	2.52
Leptobacillium	2.49
Others	16.40

G: **ORSA**

Genus	Rel. abund. (%)
Neoroussoella	5.97
Fusarium	4.06
Ascomycota_gen_Incertae_sedis	3.74
Hypocreales_gen_Incertae_sedis	3.66
Trichoderma	3.15
Chaetomium	2.62
Gibellulopsis	2.55
Thielaviopsis	2.36
Pleosporales_gen_Incertae_sedis	2.20
Nectriaceae_gen_Incertae_sedis	1.79
Others	67.90

H: **ORSS**

Genus	Rel. abund. (%)

Peniophora	23.98
Trechisporales_gen_Incertae_sedis	6.91
Fusarium	6.10
Gongronella	4.62
Ascomycota_gen_Incertae_sedis	3.48
Spiromastigaceae_gen_Incertae_sedis	3.48
Trichoderma	3.17
Talaromyces	2.57
Neoroussoella	2.29
Aspergillus	2.08
Others	41.32

Sample types: Asymptomatic, Symptomatic**; Groups: ORLA**- Okomu/Ore asymptomatic leaf samples, **ORLS**- Okomu/Ore symptomatic leaf samples, **ORRA**- Okomu/Ore asymptomatic Root samples, **ORRS**- Okomu/Ore symptomatic root samples, **ORSA**- Okomu/Ore asymptomatic soil samples and **ORSS**- Okomu/Ore symptomatic soil samples

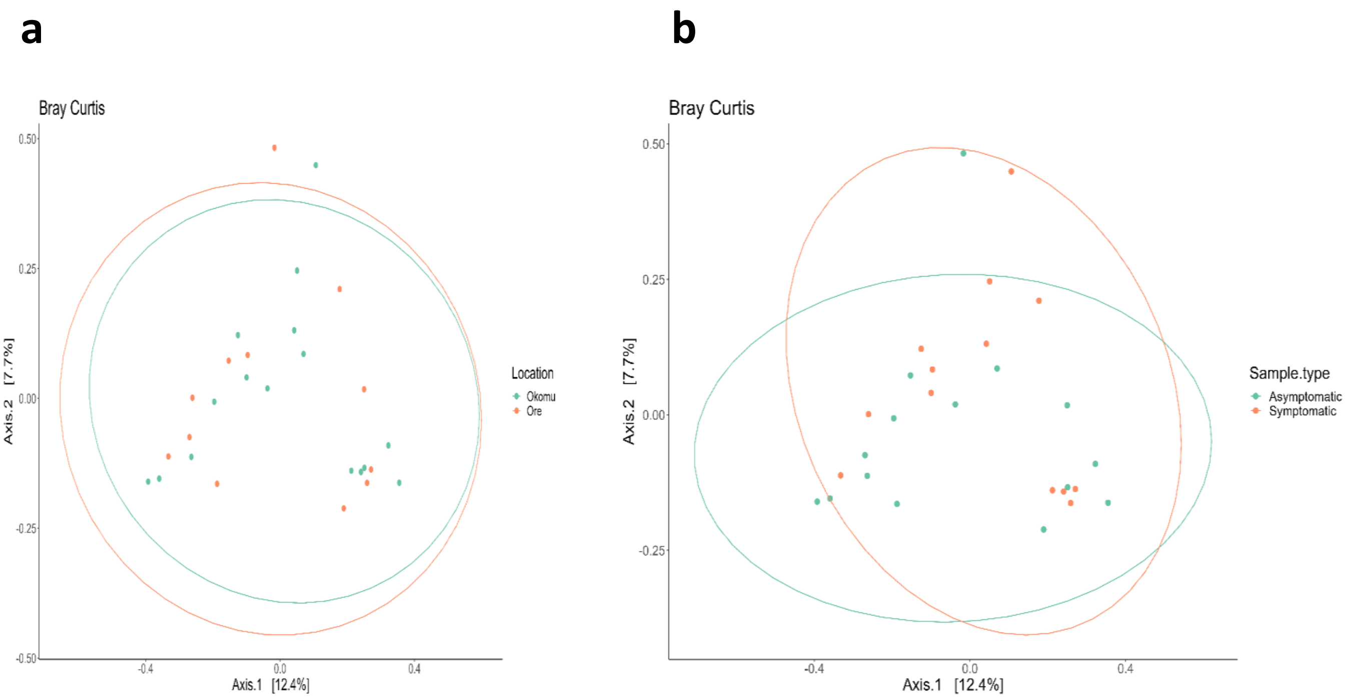

Fig. S4. Principal coordinate analysis (PCoA) based on the relative abundance of fungal ASVs. Sample categories are indicated with different colours according to (a) Location: Okomu (blue) and Ore (red) (b) Sample type: Asymptomatic (blue) and Symptomatic (red)
